# Probing Steroidal Substrate Specificity of Cytochrome P450 BM3 Variants

**DOI:** 10.3390/molecules21060760

**Published:** 2016-06-11

**Authors:** Xing Liu, Zhi-Biao Wang, Ya-Nan Wang, Jian-Qiang Kong

**Affiliations:** State Key Laboratory of Bioactive Substance and Function of Natural Medicines & Ministry of Health Key Laboratory of Biosynthesis of Natural Products, Institute of Materia Medica, Chinese Academy of Medical Sciences & Peking Union Medical College, Beijing 100050, China; seraphlx@imm.ac.cn (X.L.); wangzhibiao@live.cn (Z.-B.W.); wangyanan@imm.ac.cn (Y.-N.W.)

**Keywords:** BM3 variants, substrate specificity, 3-hydroxy-Δ^5^-steroids, 3-keto-Δ^4^-steroids, hydroxylation

## Abstract

M01A82W, M11A82W and M01A82WS72I are three cytochrome P450 BM3 (CYP102A1) variants. They can catalyze the hydroxylation of testosterone (TES) and norethisterone at different positions, thereby making them promising biocatalysts for steroid hydroxylation. With the aim of obtaining more hydroxylated steroid precursors it is necessary to probe the steroidal substrate diversity of these BM3 variants. Here, three purified BM3 variants were first incubated with eight steroids, including testosterone (TES), methyltestosterone (MT), cholesterol, β-sitosterol, dehydroepiandrosterone (DHEA), diosgenin, pregnenolone and ergosterol. The results indicated that the two 3-keto-Δ^4^-steroids TES and MT can be hydroxylated at various positions by the three BM3 mutants, respectively. On the contrary, the three enzymes displayed no any activity toward the remaining six 3-hydroxy-Δ^5^-steroids. This result indicates that the BM3 mutants prefer 3-keto-Δ^4^-steroids as hydroxylation substrates. To further verify this notion, five other substrates, including two 3-hydroxy-Δ^5^-steroids and three 3-keto-Δ^4^-steroids, were carefully selected to incubate with the three BM3 variants. The results indicated the three 3-keto-Δ^4^-steroids can be metabolized to form hydroxysteroids by the three BM3 variants. On the other hand, the two 3-hydroxy-Δ^5^-steroids cannot be hydroxylated at any position by the BM3 mutants. These results further support the above conclusion, therefore demonstrating the 3-keto-Δ^4^–steroid substrate preference of BM3 mutants, and laying a foundation for microbial production of more hydroxylated steroid intermediates using BM3 variants.

## 1. Introduction

The hydroxylation of steroids is an important enzymatic reaction in steroid metabolism and the resultant hydroxylated steroids can be used as the key-intermediates for the biosynthesis of steroid drugs with diverse therapeutic purposes [1,2,3,4,5]. Both chemical and biological approaches are thus used for the production of hydroxylated steroids [4,5,6,7,8]. Chemical synthesis suffers from the complexity of hydroxysteroids, as well as the necessity of extreme reaction conditions and the production of toxic by-products [9,10]. There are no reports, therefore, concerning the large scale production of hydroxylated steroids by chemical synthesis methods. The biotechnological production of hydroxysteroids by enzyme-catalyzed reactions or whole-cell biotransformation has made great strides over the years. Several isolated P450s and engineered strains containing heterologous P450s have been reported to hydroxylate steroids at various positions [6,7,11,12]. Among these biological methods, steroid oxidations catalyzed by engineered P450 BM3 mutants have recently gained importance for their potential use to generate rather diverse and unique hydroxylated steroids displaying important pharmacological activities [6,7,8,11].

Cytochrome P450 BM3, a well-known monooxygenase from *Bacillus megaterium*, was initially found to catalyze the NADPH-dependent hydroxylation of long-chain fatty acids [13,14]. Later, P450 BM3 has been engineered to accommodate a wide range of other substrates including short- and medium-chain fatty acid, alkanes and steroids [15,16,17]. More recently, engineered P450 BM3 variants have been reported to selectively hydroxylate steroids at various positions [6,7,8,11,18]. The first steroid-hydroxylating BM3 variant was reported in 2006 [8]. In that paper, a triple and two double mutants were shown to form mainly 16β-OH-TES [8]. Since then, different BM3 variants, like M01A82W, M11A82W and M01A82WS72I, have been described as biocatalysts for steroid hydroxylation with altered regio- and stereoselectivity [6,7,11,18,19,20]. P450 BM3 variants are thus deemed good biocatalyst candidates for the oxidative hydroxylation of steroids. The aim of previous studies was usually to identify BM3 mutants capable of hydroxylating steroids at different positions. Hence, only a few steroids, like TES [8,20], norethisterone [11,19] and progesterone [20], were used as probes to verify the hydroxylation activity of different BM3 mutants. The limited steroid substrate specificity of these BM3 mutants impedes their widespread application in the hydroxylation of steroidal precursors. It has long been known that drug-metabolising P450s with substrate promiscuity are a key factor in natural-product diversification. Therefore, it is necessary to probe the steroidal substrate diversity of these BM3 variants in order to obtain more novel hydroxylated steroid precursors. Here, the steroidal substrate specificity of three BM3 mutants M01A82W, M11A82W and M01A82WS72I, which were shown previously to hydroxylate TES and norethisterone at various positions, was explored. Specifically, the hydroxylation of eight steroids by three purified BM3 variants was performed first. The results indicated that only 3-keto-Δ^4^-steroids can be metabolized to hydroxylated metabolites. To verify this conclusion, five other steroids including three 3-keto-Δ^4^-steroids and two 3-hydroxy-Δ^5^-steroids were carefully selected and incubated with the aforementioned BM3 mutants. Detailed analysis showed only the three 3-keto-Δ^4^-steroids could be metabolized into different hydroxylated metabolites, confirming a substrate preference of BM3 mutants for 3-keto-Δ^4^-steroids. These results broaden the known steroidal substrate promiscuity of BM3 variants, thereby expanding their synthetic utility as biological catalysts.

## 2. Results and Discussion

### 2.1. Expression and Purification of BM3 Mutants

The pET28aM01A82W, pET28aM11A82W and pET28aM01A82WS72I were transformed into the *Escherichia coli* strain Transetta (DE3) for heterologous expression, respectively. 

**Figure 1:**
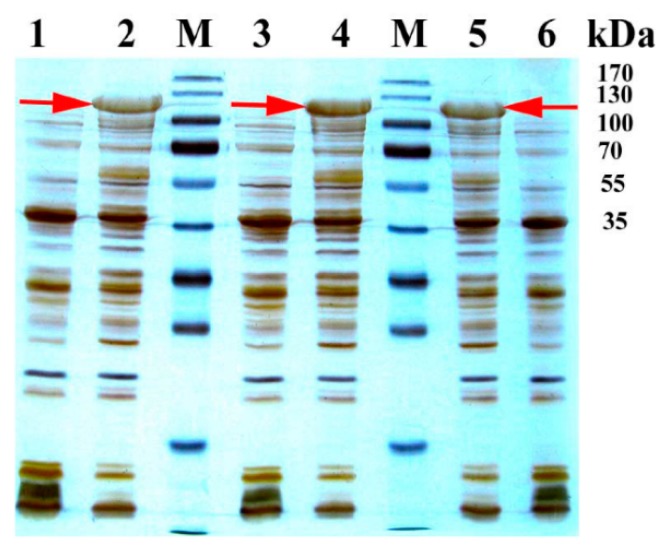
SDS-PAGE analysis of recombinant BM3 mutants. Crude protein extracts from a transformant expressing M01A82W (lane 2), M01A82WS72I (lane 4), M11A82W (lane 5) or empty vector (lanes 1, 3, 6). Lane M shows the proteins marker with the indicated molecular masses. The arrows indicated the recombinant BM3 proteins.

As depicted in Figure 1, an intense band with an apparent molecular mass of 119 kDa was determined by sodium dodecyl sulfate-polyacrylamide gel electrophoresis (SDS-PAGE) detection, suggesting the successful expression of a soluble M01A82W, M11A82W and M01A82WS72I protein. The resulting three soluble proteins were then purified to apparent homogeneity using immobilized metal affinity chromatography (IMAC).

### 2.2. Metabolism of TES by CYP102A1 Mutants

**Figure 2:**
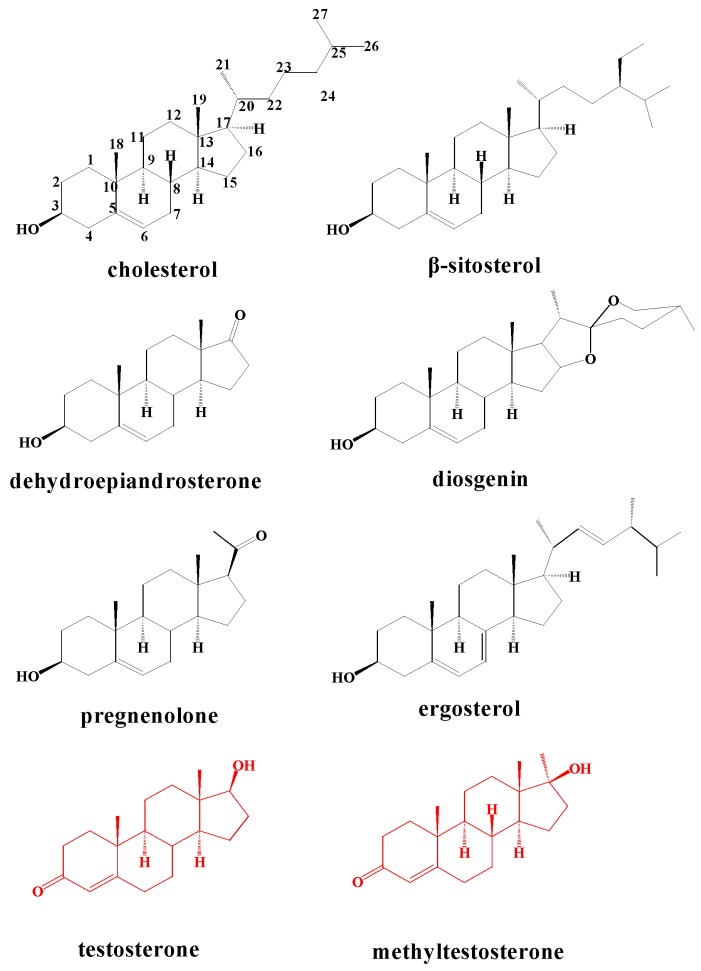
Chemical structures of the compounds used in this study.

The three BM3 mutants were successfully expressed in *E. coli*. First of all, we needed to determine whether these three purified BM3 variants possessed *in vitro* activity. Testosterone (Figure 2) has been shown to be hydroxylated by the three BM3 variants tested [6,7].

**Figure 3:**
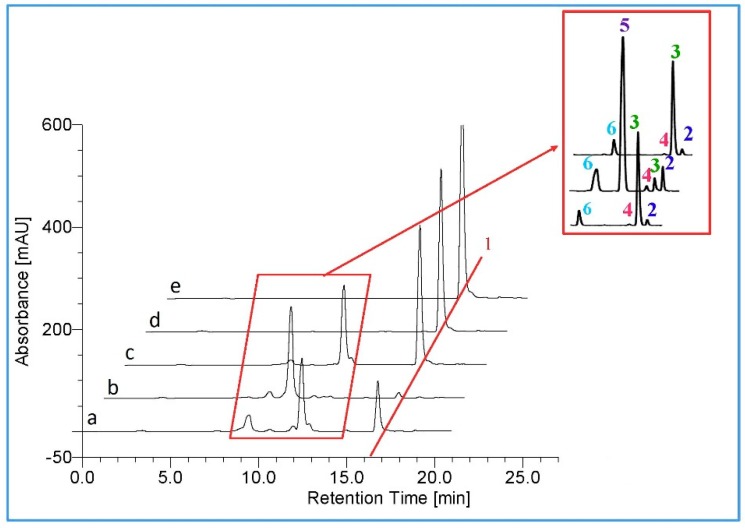
(**a**) HPLC chromatogram of testosterone; (**b**–**e**) testosterone incubation with empty vector, M01A82W, M01A82WS72I and M11A82W, respectively. Peak 1 is the testosterone standard. Peaks 2, 3, 4, 5 and 6 indicate the monohydroxy metabolites of testosterone produced by the various BM3 variants.

Hence TES was utilized as a probe for the activity characterization of the three BM3 variants in the present investigation. As illustrated in Figure 3, a total of four, five and four new metabolites were formed when TES was incubated with M01A82W, M01A82WS72I and M11A82W, respectively, thereby suggesting that the three purified BM3 mutants are active.

However, this number of products is inconsistent with previous reports [6,7]. TES was metabolized by M01A82WS72I to five metabolites, one product more than those catalyzed by the same BM3 mutant described by Venkataraman *et al.* [6]. All five metabolites were identified by LC-MS as monohydroxylated metabolites (*m*/*z* 305) (Figure S1). Product 5 was the major metabolite. For structure characterization of the major metabolite, a large-scale incubation, which contained 17.5 mL crude proteins (derived from 1000 mL cultures), 500 μM testosterone, 500 μM nicotinamide adenine dinucleotide phosphate, reduced form (NADPH), and a regeneration system (0.3 mM NADP^+^, 0.8 mM glucose 6-phosphate and 0.8 mM MgCL_2_ and 0.4 U/mL glucose 6-phosphate dehydrogenase), was performed at 28 °C for 3 h. The reaction mixture was extracted with chloroform. The chloroform extract was evaporated to dryness and the residues were resolubilized in acetonitrile. The resulting high performance liquid chromatography (HPLC) injection solution was subsequently filtered prior to preparative HPLC. The preparative major metabolite 5 was subjected to 600 MHz NMR analysis and the structure of the major metabolite was assigned as 16α-hydroxytestosterone (16α-OH-T) based on its ^1^H-NMR spectra and previously published data [6]. Details of the ^1^H spectra are tabulated in Table 1. Previous results indicated that 16α-OH-T was eluted between 15β- and 16β-OH-T. There was only one metabolite (product 6) that eluted before 16α-OH-T. Metabolite 6 was therefore presumed to be 15β-OH-T. The result presented by Venkataraman *et al* showed there were two metabolites, namely 16β- and 2β-OH-T, which eluted after 16α-OH-T. However, in the chromatogram illustrated in Figure 3, there are three monohydroxylated metabolites (2, 3 and 4) that eluted after 16α-OH-T.

To identify the exact structures of these three metabolites, the preparative separation and subsequent NMR analysis of the three metabolites were performed as mentioned above. Metabolite 2 was identified as 2β-OH-T, whereas metabolite 3 was identified as 16β-OH-T (Table 1). Since the fifth metabolite (product 4) was only formed in a tiny amount, no further efforts were made to establish its exact structure.

TES was previously reported to be metabolized by both M01A82W and M11A82W to form three monohydroxylated metabolites, namely 2β-OH-T, 15β-OH-T and 16β-OH-T [7]. In the present investigation, 2β-OH-T (product 2), 15β-OH-T (product 3) and 16β-OH-T (product 6) were also identified in the reaction mixture containing M01A82W or M11A82W. Moreover, a fourth trace metabolite 4 was detected in both the reaction mixtures. Although the exact structure of product 4 was not fully characterized due to its trace amounts, the MS data identifies it as a monohydroxylated testosterone derivative.

During the preparation of the hydroxylated metabolites, we used the crude proteins containing BM3 mutants as catalysts. To exclude any interference of *E. coli* crude proteins, a control strain harboring the empty vector pET-28a (+) was set up. As illustrated in Figure 3, no products were detected in the control *E. coli*, thereby suggesting the formation of trace product 4 is not caused by the total protein of *E. coli*, thus in this contribution, besides the previously identified metabolites, the three BM3 variants were found to hydroxylate testosterone at a new position in small amounts.

### 2.3. Metabolism of MT by CYP102A1 Mutants

**Figure 4:**
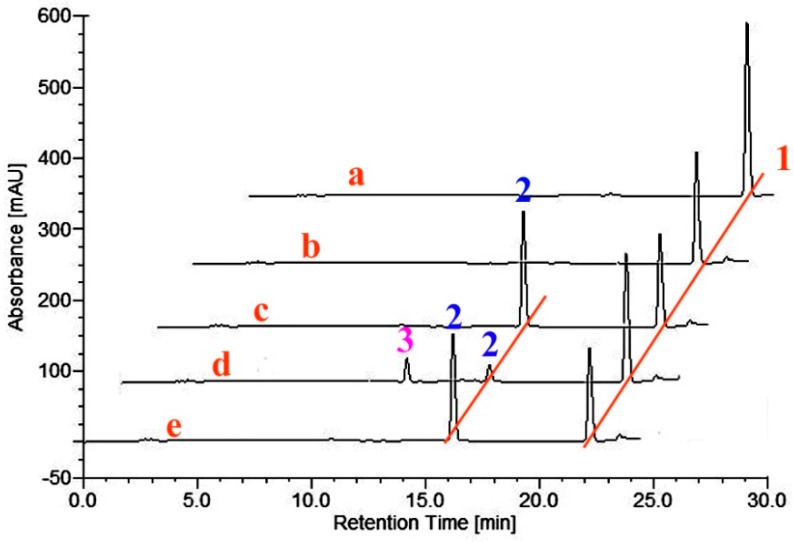
(**a**) HPLC chromatogram of methyltestosterone; (**b**–**e**) methyltestosterone incubations with empty vector, M01A82W, M01A82WS72I and M11A82W, respectively. Peak 1 is the methyl-testosterone standard; peaks 2 and 3 indicate the monohydroxy metabolites of methyltestosterone.

Having shown that the three purified BM3 mutants possess the *in vitro* ability to hydroxylate TES at different positions, the three BM3 variants were next used as probes to test different steroidal substrates. Seven steroids, including cholesterol, β-sitosterol, DHEA, diosgenin, ergosterol, pregnenolone and MT (Figure 2), were incubated with the three P450 BM3 mutants, respectively. The results indicated that new metabolites were formed after incubation of 200 μM MT in the presence of the three variants of CYP102A1 (Figure 4). 

**Figure 5:**
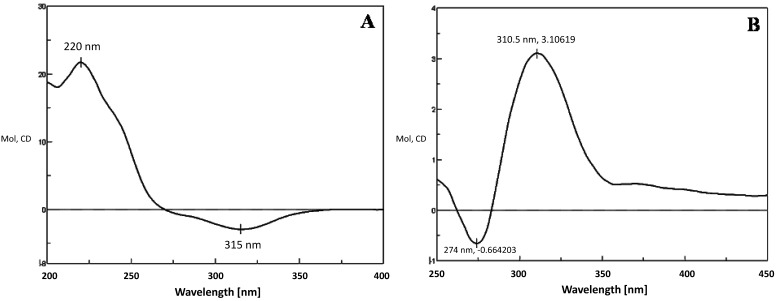
CD spectrum of 16β-OH-MT without Mo_2_(OAc)_4_ (**A**) or mixed with Mo_2_(OAc)_4_ (**B**).

As illustrated in the chromatogram obtained after incubation of BM3 mutant M01A82W with MT, one major metabolite was produced (Figure 4). The mass spectrum of this metabolite showed a [M + H]^+^ peak at a *m*/*z* value of 319.29 (Figure S2), consistent with the introduction of a hydroxyl group into MT. Its structure was determined on the basis of a combination of ^1^H-NMR, ^13^C-NMR, HMQC, HMBC and CD assays. Detailed ^1^H-NMR and ^13^C-NMR data are given in Table 2. As shown in the HMBC, there is only one oxygenated methylene signal (δH 3.63, dd), which has long-range correlation both to 20-CH3 and 14-C, so the newly hydroxylated methylene is determined to be C-16. The OH group was determined to be in a β-orientation from the CD experiment. After the addition of Mo_2_(OAc)_4_, a significant positive Cotton effect was observed at 310 nm (Figure 5), thereby assigning this metabolite as 16β-OH-MT according to the literature analysis [21,22].

**Figure 6:**
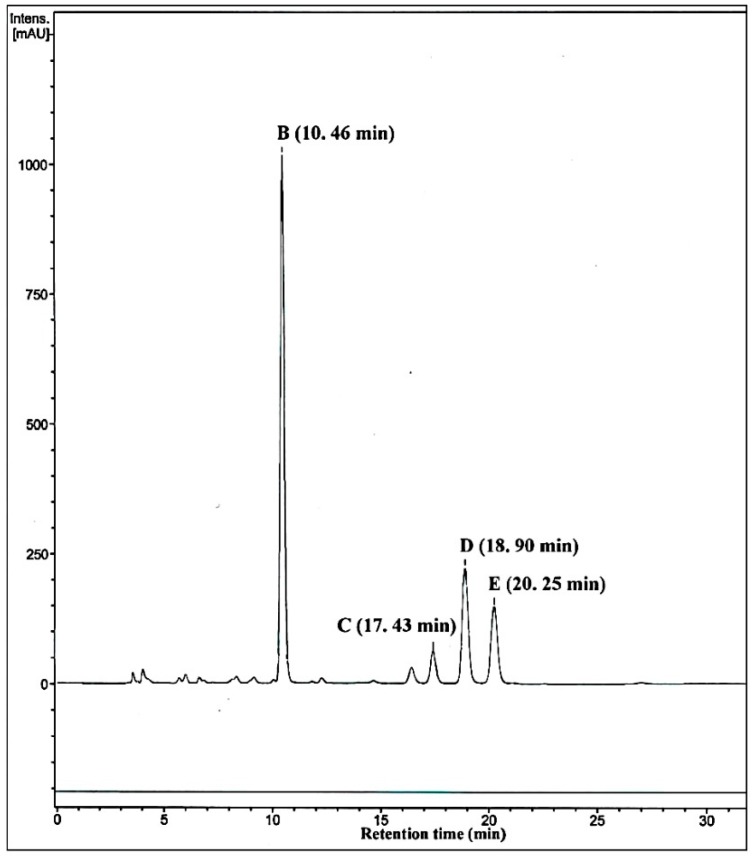
HPLC profile of a large scale incubation of methyltestosterone with M01A82WS72I. The four metabolites eluted at 10.46 (B), 17.43 (C), 18.90 (D) and 20.25 (E) min, respectively.

M1182W also converted MT to form mainly 16β-OH-MT (Figure 4). However, M01A82WS72I can metabolize MT to form several new metabolites (Figure 4). Besides the two major metabolites (peaks 2 and 3), there were several metabolites formed in trace amounts that eluted between products 2 and 3 (Figure 4). A HPLC-NMR coupling technology was therefore applied, allowing rapid and detailed structural characterization of the reaction mixture catalyzed by M01A82WS72I. A large-scale incubation (25 mL) of M01A82WS72I with MT was performed. The resulting reaction mixture was subsequently injected to the HPLC-NMR system for analysis. As illustrated in Figure 6, four new metabolites with retention times (*R*_t_) of 10.46 (B), 17.43 (C), 18.90 (D) and 20.25 (E) min were obtained after incubation of M01A82WS72I with MT. Figure S3 shows the ESI mass spectrum of the four MT metabolites which generated abundant [M + H]^+^ ions at a *m*/*z* value of 319. This pseudomolecular ion was in accordance with the hypothesis of an hydroxylated metabolite of MT [23]. The metabolites eluted at 10.46, 18.90 and 20.25 min were assigned to 16α-OH-MT, 16β-OH-MT and 2β-OH-MT based on the NMR spectra and ^1^H and ^13^C chemical shifts. The assignment of 16β-OH-MT is the same as described above. The structural identification of 2β-OH-MT and 16α-OH-MT was described as follows (Table 2):

16α-OH-MT: in the HMBC, there is only one oxygenated methylene signal (δ_H_ 4.18, dd), which has long-range correlations to 20-CH3, 13-C and 14-C, so the newly hydroxylated methylene is determined to be C-16. The oxygenated methane signal (δ_H_ 4.18) is at lower field than in 16β-OH-MT (δ_H_ 3.63), indicating it is sterically affected by 17-CH3, which is an α-orientation. Besides, its HPLC retention time is shorter than that 16β-OH-MT, which together determined the 16-OH to be in the α-orientation (Table 2).

2β-OH-MT: the final quantity obtained was not far from the detection limit of the ^13^C-NMR instrument used. The ^1^H-NMR spectrum was recorded to demonstrate the purity of the product and to confirm its structure. In the HMBC, 4-H (δ_H_ 5.68) is long-ranged correlated to C-2, C-6, and C-10. In the 3 signal of the 3-carbonyl, the δ_C_ of C-2 should be the largest (δ_C_ 69.0). In the HMQC, it is directly correlated to the only one oxygenated methylene signal (δH 4.15, dd). All these clues suggest the hydroxyl group is attached to C-2.The narrow-wide doublet-doublet shape of H-2 indicated that this proton was axial. In other words, the orientation of the hydroxyl was on the β-face [24].

Details of the ^1^H- and ^13^C-NMR spectra of 2β-OH-MT and 16α-OH-MT are also tabulated in Table 2.

The metabolite C eluted at 17.43 min has not been well-characterized due to the trace amount available. The three BM3 mutants did not metabolize any of the other six steroids tested.

### 2.4. Metabolism of 3-keto-Δ^4^-steroids by CYP102A1 Mutants

**Figure 7:**
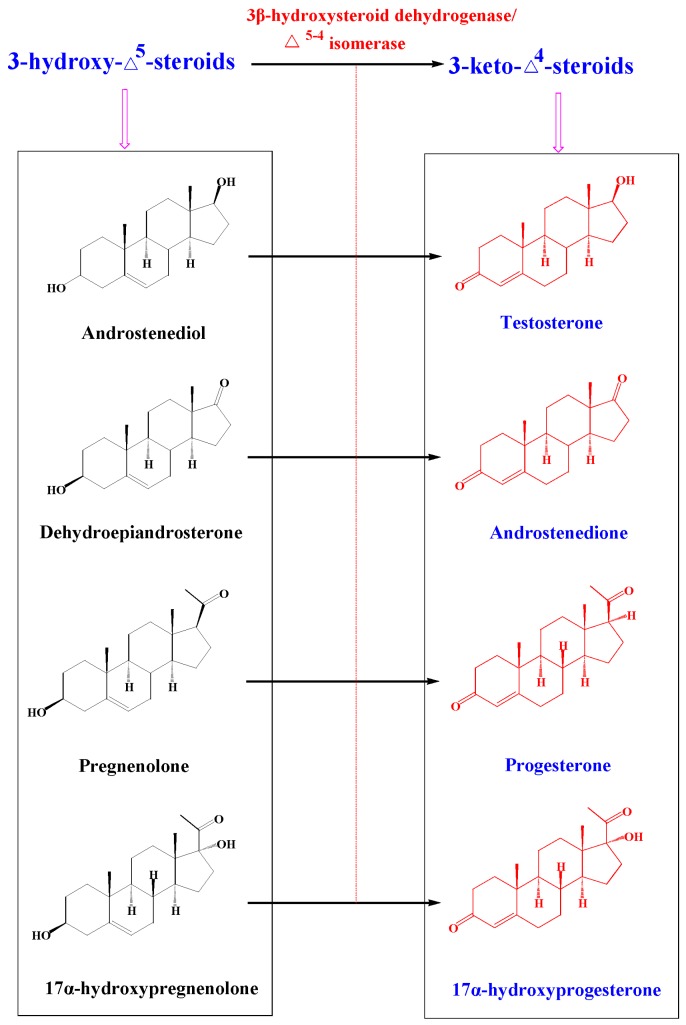
3-keto-Δ^4^-steroids and 3-hydroxy-Δ^5^-steroids used in this study.

Structural analysis reveals that TES and MT are two 3-keto-Δ^4^-steroids, whereas the other six steroids not hydroxylated by BM3 variants are all 3-hydroxy-Δ^5^-steroids. Hence, we presumed that the three BM3 mutants preferred 3-keto-Δ^4^-steroids as substrates. To verify the hypothesis, five steroids, including two 3-hydroxy-Δ^5^-steroids and three 3-keto-Δ^4^-steroids, were further carefully selected to incubate with the three BM3 mutants (Figure 7). The two 3-hydroxy-Δ^5^-steroids include 17α-hydropregnenolone and androstenediol, while the other three 3-keto-Δ^4^-steroids are androstenedione, progesterone and 17α-hydroprogesterone (Figure 7). The five compounds, together with the aforementioned three steroids DHEA, pregnenolone and TES, can form four pairs of structurally similar compounds, namely DHEA and androstenedione, pregnenolone and progesterone, 17α-hydropregnenolone and 17α-hydroprogesterone, as well as androstenediol and TES (Figure 7).

**Figure 8:**
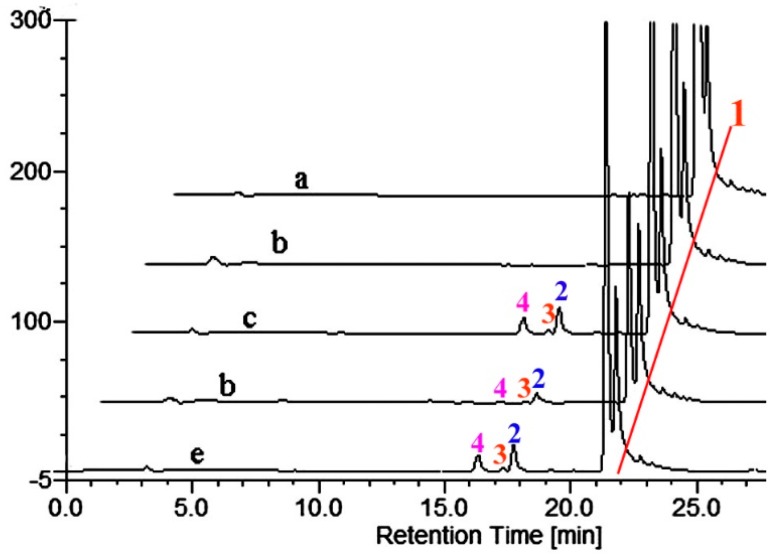
(**a**) HPLC chromatogram of progesterone; (**b**)–(**e**) progesterone incubations with empty vector, M01A82W, M01A82WS72I and M11A82W, respectively. Peak 1 is a progesterone standard; peaks 2, 3 and 4 are three monohydroxylated metabolites of progesterone.

**Figure 9:**
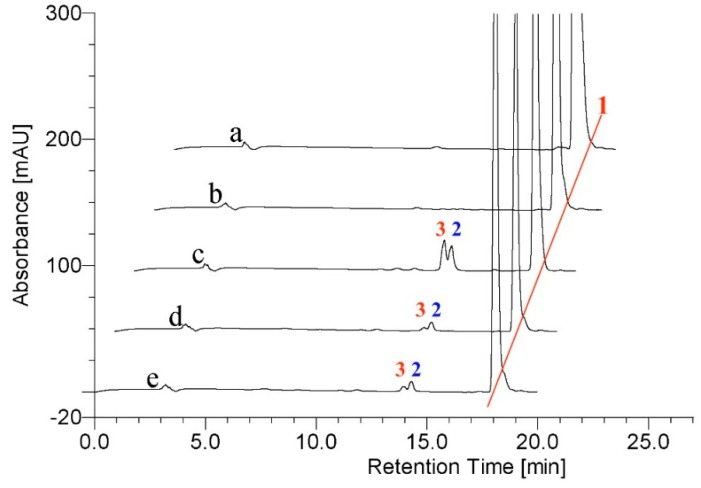
(**a**) HPLC profile of 17α-hydroxyprogesterone; (**b**–**e**) 17α-hydroxyprogesterone incubations with empty vector. M01A82W. M01A82WS72I and M11A82W, respectively. Peak 1 shows a 17α-hydroxyprogesterone standard; peaks 2 and 3 show the two monohydroxylated metabolites of 17α-hydroxyprogesterone.

**Figure 10:**
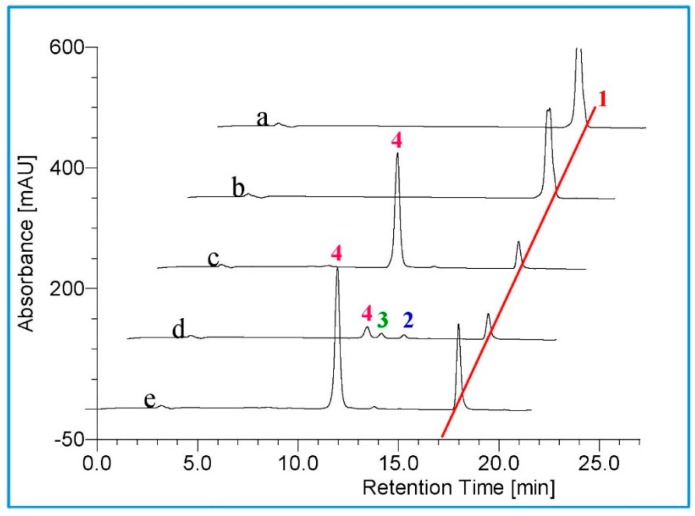
(**a**) HPLC chromatogram of androstenedione; (**b**–**e**) androstenedione incubations with empty vector, M01A82W, M01A82WS72I and M11A82W, respectively. Peak 1 shows the androstenedione standard; peaks 2, 3 and 4 are three monohydroxylated metabolites of androstenedione.

Actually, the four 3-hydroxy-Δ^5^-steroids are substrates of the respective Δ^4^-3-keto configuration. The biosynthesis of DHEA from androstenedione, pregnenolone from progesterone, 17α-hydropregnenolone from 17α-hydroprogesterone, and androstenediol from TES occur under the action of 3β-hydroxysteroid dehydrogenase/Δ^5−4^ isomerase (3β-HSD, EC 1.1.1.145) (Figure 7). These structurally similar compounds were therefore selected to incubate with the three purified BM3 mutant proteins. HPLC profiles showed the BM3 mutants can metabolize the four 3-keto-Δ^4^-steroids to form various metabolites (Figure 3, Figure 4, Figure 6, Figure 8, Figure 9 and Figure 10). However, there are no new metabolites after incubation of the 3-hydroxy-Δ^5^-steroids with the three purified enzymes (Figures S5, S7, S9 and S10).

As illustrated in Figure 8, all three BM3 mutants M01A82W, M01A82WS72I and M11A82W can convert progesterone to form three new metabolites (2, 3 and 4). These new metabolites were identified by MS as monohydroxylated products (*m*/*z* 331, Figure S4). However, the further structural characterizations of these compounds were not performed due to their trace amount. On the other hand, when pregnenolone, a Δ^5^–3-hydroxy configuration of progesterone, was added into reaction systems containing varied BM3 variants, no new products were detected (Figure S5).

17α-Hydroxyprogesterone is also a 3-keto-Δ^4^-steroid. As shown in Figure 9, all the three BM3 enzymes can metabolize 17α-hydroxyprogesterone to form two monohydroxylated metabolites with an *m*/*z* value of 347 (Figure S6). The concentrations of the two metabolites are too low to perform structural analyses. However, when 17α-hydroxypregnenolone, a substrate of 17α-hydroxyprogesterone, was incubated with the three BM3 proteins, no new metabolites were appeared in the HPLC profiles (Figure S7).

Androstenedione and DHEA are structurally similar compounds. When the two steroids reacted with the three BM3 enzymes, two opposite results were not unexpected. Specifically, androstenedione can be hydroxylated by the three BM3 proteins at varied positions, which can be verified by the corresponding HPLC profile (Figure 10) and ESI-MS results (Figure S8). On the contrary, DHEA has no reactivity with any of the three BM3 variants (Figure S9). As indicated in Figure 10, M01A82W can hydroxylate androstenedione at one position. The sole metabolite was subsequently assigned to be 1α-hydroxyandrostenedione based on the combination of LC-MS and NMR analyses. The NMR data details are summarized in Table 3. M11A82W can add a hydroxyl group to androstenedione at the same position. Besides hydroxylating at the 1β position, M01A82WS72I is able to hydroxylate androstenedione at two other positions. The exact structures of two monohydroxylated metabolites had never been well-characterized due to their trace amount.

Androstenediol is the Δ^5^–3-hydroxy configuration of testosterone. Androstenediol was therefore incubated with the three BM3 mutants. As expected, the incubation of androstenediol with the BM3 variants did not result in any new products (Figure S10).

The hydroxylation of steroids is an important enzymatic reaction in the course of preparation of oxysterols and saponins with diverse pharmacological activities. As biocatalysts capable of hydroxylating steroids, bacterial P450 BM3 mutants are considered to have a very broad application prospects. However, the limited substrate diversity mentioned previously hinders their application in the preparation of hydroxylated steroids. In the present investigation, we investigated the oxidation effects of three BM3 mutants on multiple steroid substrates. The P450 BM3 variants chosen for this experiment included M01A82W, M11A82W and M01A82WS72I, which had been shown to display regio- and stereoselective hydroxylation activity towards a few steroid probes [6,7]. From the results, we revealed that these P450 BM3 mutants preferred 3-keto-Δ^4^-steroids as substrates. To our knowledge, this is the first report about the substrate preference of P450 BM3 variants, which lays a foundation for a structure-activity relationship study of bacterial P450 BM3s.

## 3. Experimental Section

### 3.1. Substrates, Chemicals and Enzymes

Materials used in this study were as follows: a total of 13 steroids were used as substrates in the enzyme assays. The details of the sources of these steroidal substrates are summarized in Supplementary Materials Table S1; Restriction enzymes (Takara Shuzo Co. Ltd., Kyoto, Japan) and KOD-Plus-Neo DNA polymerases (Toyobo Co. Ltd., Osaka, Japan) were applied to construct expression vectors; Ni-Sepharose (Invitrogen, Carlsbad, CA, USA) was used for protein purification; Fast Mutagenesis System kit was used for the site-directed mutagenesis of BM3 variants (TransGen Biotech Co. Ltd., Beijing, China). All other chemicals used in this study were of analytical grade.

### 3.2. Strains and Plasmids

Prokaryotic expression vector pET-28a (+), which was used for heterologous expression, was purchased from Novagen (Madison, WI, USA). The CYP102A1 mutants M01A82W (R47L/A82W/F87V/L188Q/E267V) and M11A82W (R47L/E64G/F81I/A82W/F87V/E143G/L188Q/E267V/G415S) [7] were synthesized and cloned into expression vector pET-28a (+) by Taihe Biotechnology Co. Ltd. (Beijing, China) One of the resulting expression vector pET28aM01A82W was used either for heterologous expression of M01A82W, or as the template for the third BM3 mutant M01A82WS72I [6]. The *E. coli* strain Trans1-T1 and Transetta (DE3) (TransGen Co. Ltd., Beijing, China) were used as a bacterial host for recombinant plasmid amplification and enzyme expression, respectively. The strain was grown in Luria-Bertani medium(10 g/L Bacto-Tryptone, 5 g/L Bacto-yeast extract, 10 g/L NaCl) or induced in TB medium (12 g/L Bacto-Tryptone, 24 g/L Bacto-yeast extract, 4 mL glycerol, 72 mM K_2_HPO_4_, 17 mM KH_2_PO_4_), supplemented with appropriate antibiotics, *i.e.*, kanamycin (30 mg/mL) and chloromycetin (34 mg/mL) for selection.

### 3.3. Site-Directed Mutagenesis

Site-directed mutagenesis was performed to obtain the third BM3 mutant M01A82WS72I as described previously [25]. Specifically, the S72I mutation were introduced into M01A82W by PCR-based amplification of the entire pET28aM01A82W expression plasmid using two mutated oligonucleotide primers, each complementary to the opposite strand of the vector. The sequence of the forward primer for the mutation was 5′-TTTGATAAAAACTTAA***T***TCAAGCGCTT-3′, with the altered residue shown in bold italics. The reverse primer for this position was 5′-***A***TTAAGTTTTTATCAAAGCGTGATTCA-3′. All components necessary for PCR-based mutagenesis, contained in the Fast Mutagenesis System kit, were used according to the manufacturer’s instructions. The mutants were confirmed by sequencing and those plasmids with target substitutions and without other unwanted mutations were retained.

### 3.4. Expression and Purification of BM3 Mutants

The expression plasmids pET28aM01A82W, pET28aM11A82W and pET28aM01A82WS72I were transformed into the expression host strain Tran*s*etta (DE3) for heterologous expression of the three BM3 variants. Strains inoculation and genes induction were performed as described previously [25,26,27,28]. Briefly, a single transformant containing pET28aM01A82W, pET28aM11A82W or pET28aM01A82WS72I was used to start a 10 mL LB culture with chloromycetin (34 mg/mL) and kanamycin (30 mg/mL) at 37 °C and 200 rpm. The starter culture was then used to inoculate 100 mL TB medium for continuous culture until OD_600_ reached 0.6 at the same antibiotic concentrations as above. Subsequently, isopropyl β-D-thiogalactopyranoside (IPTG) was added to induce the heterologous expression of M01A82W, M11A82W and M01A82WS72I at the final concentration of 0.1 mM. After induction culture at 20 °C for 20 h, the induced cells were harvested by centrifugation (10,000 *g*, 5 min) at 4 °C. The resultant cell pellets (derived from 1 mL culture) were analyzed via SDS-PAGE to confirm the presence of recombinant M01A82W, M11A82W or M01A82WS72I, first and then, the rest cell pellets were prepared for protein purification.

For enzyme purification, all steps were performed at 4 °C. First, *E. coli* cells were washed and resuspended in lysis buffer (50 mM potassium phosphate, pH7.4). Cells were then lysed with a high-pressure homogenizer (800 bar, 3 passes), after which 1 U/mL DNaseI was added, and then the homogenate was incubated at 4 °C for approximately 2 h. After centrifugation at 12,000 g for 30 min, the supernatant was passed through a 0.45 μm pore-size filter to remove *E. coli* cell debris and other contaminants, and then loaded onto a pre-equilibrated (20 mM sodium phosphate buffer containing 10–50 mM imidazole and 300 mM NaCl, pH 8.0) to remove non-specifically bound proteins, after which an elution buffer (20 mM sodium phosphate containing 200 mM imidazole and 300 mM NaCl, pH 8.0) was used to elute the His_6_-tagged protein. To remove small molecules such as imidazole, dialysis was performed. A semipermeable membrane with a molecular weight cutoff of 30 kDa was selected and approximately 20 mL protein sample was dialyzed against 1 L dialysis buffer (10 mM sodium phosphate, pH 8.0) for 4 h at 4 °C with four changes of dialysis buffer. Proteins were then either stored at −80 °C or used directly.

### 3.5. Metabolism of Steroids by CYP102A1 Mutants

Hydroxylation activity of purified M01A82W, M11A82W or M01A82WS72I was determined by measuring the formation of hydroxylated derivatives from steroid substrates by *in vitro* reactions. Unless specified otherwise, the final volume of the reaction mixture was 200 μL, with a steroid substrate concentration of 200 μM. The reactions were initiated by addition of an NADPH regenerating system (final concentrations of 0.2 mM NADPH, 0.3 mM glucose 6-phosphate, and 0.4 unit/mL glucose-6-phosphate dehydrogenase) using an enzyme-coupled method [29]. The reaction was allowed to proceed for 3 h at 25 °C and terminated by the addition of 100 μL of chloroform. Precipitated protein was removed by centrifugation (16,200 *g*, 5 min), and the organic layers were evaporated using a vacuum pump. The formation of hydroxylated steroid derivatives was unambiguously determined by a combination of by HPLC-UV, HPLC-MS, ^1^H- and ^13^C-NMR and LC-SPE-NMR.

HPLC-UV was performed on a LaCrom elite L-2000 HPLC system (Hitachi, Toyokawa, Japan) using a C18 column (YMC-Pack ODS-A (5 μm, 12 nm, 250 × 4.6 mm)). Chromatographic condition was as follow. The mobile phase consisted of deionized water-trifluoroacetic acid (A, 99.95%:0.05%, *v*/*v*) and acetonitrile (B) in gradient mode as follows: from 1 to 15 min, linear increase from 20% to 60% B; from 15 to 20 min, linear increase to 100% B. The flow rate was kept at 1.0 mL/min and the column temperature was maintained at ambient. The sample injection volume was 50 μL. The DAD detection was performed in the range of 210–284 nm. LC-MS and NMR analyses were performed as previously reported [26,27,30]. LC-SPE-NMR experiments were performed using an Agilent 1260 series HPLC (Agilent, Palo Alto, CA, USA) interfaced with an AVANCE III HD 600 MHz spectrometer (Bruker, Fallanden, Switzerland). The chromatographic separation was performed using an YMC-Pro C18 column (5 μm, 12 nm, 250 × 4.6 mm) with an isocratic elution of 65% water-trifluoroacetic acid (A, 99.95%:0.05%, *v*/*v*) and 35% acetonitrile (B) at a flow rate of 0.8 mL/min. The column temperature was maintained at 30 °C. UV spectra were recorded from 190 to 400 nm. Varied hydroxylated metabolites of steroids were enriched using the on-line solid-phase extraction (SPE) add-on. The resulting enriched metabolites were then structurally characterized by NMR analysis at 600 MHz for ^1^H-NMR and 150 MHz for ^13^C-NMR using the solvent CDCl_3_. Chemical shifts (δ) are given in ppm, coupling constants (*J*) are given in Hertz (Hz).

## 4. Conclusions

Bacterial P450 BM3 mutants were reported to display altered regio- and stereoselectivities in hydroxylations of a few steroids. P450 BM3 mutants were therefore deemed to be promising candidates for biocatalysis of steroids. Hence, it is necessary to probe the steroidal substrate diversity of these BM3 variants with the aim of obtaining more hydroxylated steroids precursors. In the present investigation, a total of 13 steroids were used as substrates to probe the hydroxylation capacity of three representative BM3 mutants. The results revealed that the three BM3 proteins were indeed able to metabolize 3-keto-Δ^4^-steroids to monohydroxylated metabolites. On the contrary, the three BM3 mutants had no any oxidative activity on 3-hydroxy-Δ^5^-steroids. These results suggest a substrate preference of BM3 mutants towards 3-keto-Δ^4^-steroids. These results broaden our knowledge of the steroid substrate promiscuity of BM3 variants, thereby expanding their synthetic utility as biological catalysts.

## Figures and Tables

**Table 1:** ^1^H and ^13^C-NMR data for 16β-OH-T, 16α-OH-T and 2β-OH-T (600 MHz for ^1^H-NMR and 150 MHz for ^13^C-NMR, D_2_O, *δ* in ppm).

Position	16β-OH-T		16α-OH-T *	2β-OH-T	
	δ_C_	δ_H_	δ_H_	δ_C_	δ_H_
1	35.7	1.50–1.42 (m, 1H); 1.88–1.84 (m, 1H)	1.50–1.47 (m, 1H); 1.80–1.77 (m, 1H)	41.3	1.63–1.56 (m, 1H); 2.05 (m, 1H)
2	34	2.06–2.00 (m, 1H); 2.34–2.27 (m, 1H)	2.04–2.00 (m, 1H); 2.35–2.29 (m, 1H)	68.6	4.00–3.95 (m, 1H)
3	199.5			199.4	
4	124	5.73 (s, 1H)	5.72 (s, 1H)	120.3	5.63 (s, 1H)
5	170.9			172	
6	32.7	2.28–2.26 (m, 1H); 2.37–2.34 (m, 1H)	2.29–2.25 (m, 1H); 2.42–2.37 (m, 1H)	30.3	2.19–2.16 (m, 1H); 2.40–2.37 (m, 1H)
7	31.7	1.02 -0.97 (m, 1H); 1.73–1.68 (m, 1H)	1.00–0.98 (m, 1H); 1.73–1.68 (m, 1H)	32.5	0.85 (m, 1H); 1.87–1.84 (m, 1H)
8	35	1.50–1.42 (m, 1H)	1.50–1.47 (m, 1H)	34.1	1.63–1.56 (m, 1H)
9	54.1	0.97–0.93 (m, 1H)	0.99–0.96 (m, 1H)	51.1	1.37–1.34 (m, 1H)
10	42.4			43.3	
11	20.4	1.31–1.27 (m, 1H); 1.35–1.31 (m, 1H)	1.47–1.44 (m, 2H)	22.1	1.63–1.56 (m, 1H); 1.50–1.44 (m, 1H)
12	37	1.06–1.03 (m, 1H); 1.67–1.63 (m, 1H)	1.04–1.00 (m, 1H); 1.43–1.40 (m, 1H)	35.6	1.03 (m, 1H); 1.78–1.72 (m, 1H)
13	38.7			36.7	
14	47	0.81–0.79 (m, 1H)	0.81–0.79 (m, 1H)	50.1	0.85 (m, 1H)
15	35	1.15–1.10 (m, 1H); 1.93–1.88 (m, 1H)	1.83–1.81 (m, 1H); 1.06–1.04 (m, 1H)	23.5	1.50–1.44 (m, 1H); 1.34 (m, 1H)
16	70	3.39–3.36 (m, 1H)	4.17–4.13 (m, 1H)	29.5	1.85–1.81 (m, 1H); 1.43–1.39 (m, 1H)
17	80.7	4.19 (m, 1H)	3.50 (d, *J* 5.7, 1H)	80.3	3.47–3.41 (m, 1H)
18	11.9	0.86 (s, 3H)	0.84 (s, 3H)	11.8	0.67 (s, 3H)
19	17.4	1.20 (s, 3H)	1.18 (s, 3H)	22.4	1.14 (s, 3H)

* No ^13^C-NMR analysis of 16α-OH-T was performed.

**Table 2:** NMR data for 16α-OH-MT, 16β-OH-MT and 2β-OH-MT (600 MHz for ^1^H-NMR and 150 MHz for ^13^C-NMR, D_2_O, *δ* in ppm) obtained via HPLC-NMR.

Position	16α-OH-MT			16β-OH-MT			2β-OH-MT		
	δ_C_	δ_H_	HMBC	δ_C_	δ_H_	HMBC	δ_C_	δ_H_	HMBC
1	35.8	1.87, m	C2, C3, C10, C19	36.8	2.16, m	C2, C3	41.6	1.61–1.68, m	C2, C9, C10, C19
2	33.2	2.25, m; 2.42, m	C1, C3, C4, C10	33.9	2.25, m; 2.43, m	C1, C3, C4	69.8	4.15–4.18, m	C1, C3
3	200.9			202.4			201.6		
4	122.7	5.68, s	C3, C5	124.1	5.68,s	C3, C5	120.5	5.69, s	C2, C6, C10
5	173.7			175.1			174.3		
6	33.2	2.28, m; 2.46, m	C4, C5, C7	34.7	2.29, m; 2.47, m	C4, C5, C7	32.7	2.39–2.45, m;2.51–2.59, m	C5, C7, C10
7	31.4	1.38, m; 1.81, m	C6, C8, C9	33.1	1.03, m;1.87, m	C6, C8, C9	33.9	1,31, m	C5, C9
8	35.8	1.60, m	C6, C8, C9	37.2	1.69, m	C7, C9	35.7	1.61–1.68, m	C9, C10, C14, C15
9	35.8	0.93, m	C8, C10, C11, C12, C19	55.4	0.91, m	C8, C10, C11, C19	51.5	0.93, m	C8, C10
10	38.6			37.2			42.4		
11	19.8	1.46, m; 1.51, m	C9, C12	21.6	1.48, m;1.53, m	C9, C12	27.1	1.51–1.56, m; 1.61–1.68, m	C9, C12
12	31.5	1.00, m; 1.67, m	C9, C18	33.5	0.94, m; 1.60, m	C9, C13, C18	37.8	1.26–1.28, m	C18
13	45.8			46.0			47.2		
14	47.9	1.41, m	C13, C15, C16, C18	47.9	1.31, m	C13, C15, C16, C18	49.9	0.95–1.03, m	C8, C9, C15
15	32.4	1.35, m; 2.06, m	C13, C14, C16	35.8	0.99, m;2.06m	C13, C14, C16	24.2	1.28–1.30, m; 1.51–1.56, m	C14, C16
16	79.1	4.15, m	C14, C15, C17	78.3	3.58, m	C15, C17, C20	26.1	1.80–188, m	C15
17	83.4			79.8			82.0		
18	13.4	0.89, s	C12, C13, C17	14.2	0.87, s	C12, C13, C17	14.7	0.87, s	C12, C13, C17
19	16.2	1.21, s	C1, C9	17.7	1.22, s	C9, C10	23.0	1.21, s	C13, C16, C17
20	16.7	1.11, s	C13, C16, C17	24.2	1.07, s	C13, C16, C17	23.5	1.16, s	C5, C9, C10

**Table 3:** NMR data for 1α-OH-androstenedione (600 MHz for ^1^H-NMR and 150 MHz for ^13^C-NMR, DMF, *J* in Hz, δ in ppm).

Position	1α-OH-Androstenedione	
	δ_C_	δ_H_
1	74.77	3.81 (t, *J* = 8.6 Hz, 1H)
2	35.73	2.40–2.36 (m, 1H) 2.42 (dd, *J* = 14.0 Hz, 5.0, 1H)
3	198.22	
4	123.55	5.57 (s, 1H)
5	170.74	
6	32.34	2.26–2.19 (m, 2H)
7	31.59	0.94-0.88 (m, 1H) 1.88–1.85 (m, 1H)
8	33.96	1.58–1.53 (m, 1H)
9	46.63	1.68–1.63 (m, 1H)
10	38.92	
11	20.3	1.43–1.34 (m, 1H )1.58–1.53 (m, 1H)
12	31.85	1.02–0.95 (m, 1H) 1.74 (ddd, *J* = 22.3, 11.2, 3.5 Hz, 1H)
13	44.81	
14	54.24	
15	31.24	1.43–1.34 (m, 1H) 1.96–1.92 (m, 1H)
16	35.52	2.13–2.07 (m, 1H) 2.36–2.30 (m, 1H)
17	218.95	
18	14.16	0.85 (s, 3H)
19	16.7	1.15 (s, 3H)

## Supplementary Materials

Supplementary materials can be accessed at: http://www.mdpi.com/1420-3049/21/6/760/s1.
