# Supplementary material for: Probing Steroidal Substrate Specificity of Cytochrome P450 BM3 Variants
Source: Molecules. 2016 Jun 11;21(6):760. doi: 10.3390/molecules21060760 (PMC6273762; doi:10.3390/molecules21060760)
Supplement: Supplementary file 1 [file molecules-21-00760-s001.pdf]

Supplementary Materials: Probing Steroidal Substrate Specificity of Cytochrome P450 BM3 Variants

Xing Liu, Zhi-Biao Wang, Ya-Nan Wang and Jian-Qiang Kong

Table S1. Compounds used in this study.

No.	Steroid	Structure	Source
1	cholesterol	3-hydroxy-Δ^5–steroid	Sigma-Aldrich, St. Louis, MO, USA
2	β-sitosterol	3-hydroxy-Δ^5–steroid	National Institutes for Food and Drug Control, Beijing, China
3	diosgenin	3-hydroxy-Δ^5–steroid	Sigma-Aldrich, St. Louis, MO, USA
4	ergosterol	3-hydroxy-Δ^5–steroid	National Institutes for Food and Drug Control, Beijing, China
5	methyltestosterone	3-keto-Δ^4–steroid	National Institutes for Food and Drug Control, Beijing, China
6	androstenediol	3-hydroxy-Δ^5–steroid	Alke Reagent, Chengdu, China
7	testosterone	3-keto-Δ^4–steroid	J&K Chemical Ltd., Shanghai, china
8	dehydroepiandrosterone	3-hydroxy-Δ^5–steroid	Alke Reagent, Chengdu, China
9	androstenedione	3-keto-Δ^4–steroid	National Institutes for Food and Drug Control, Beijing, China
10	pregnenolone	3-hydroxy-Δ^5–steroid	National Institutes for Food and Drug Control, Beijing, China
11	progesterone	3-keto-Δ^4–steroid	National Institutes for Food and Drug Control, Beijing, China
12	17α-hydroxypregnenolone	3-hydroxy-Δ^5–steroid	Sigma-Aldrich, St. Louis, MO, USA
13	17α-hydroxyprogesterone	3-keto-Δ^4–steroid	J&K Chemical Ltd., Shanghai, China

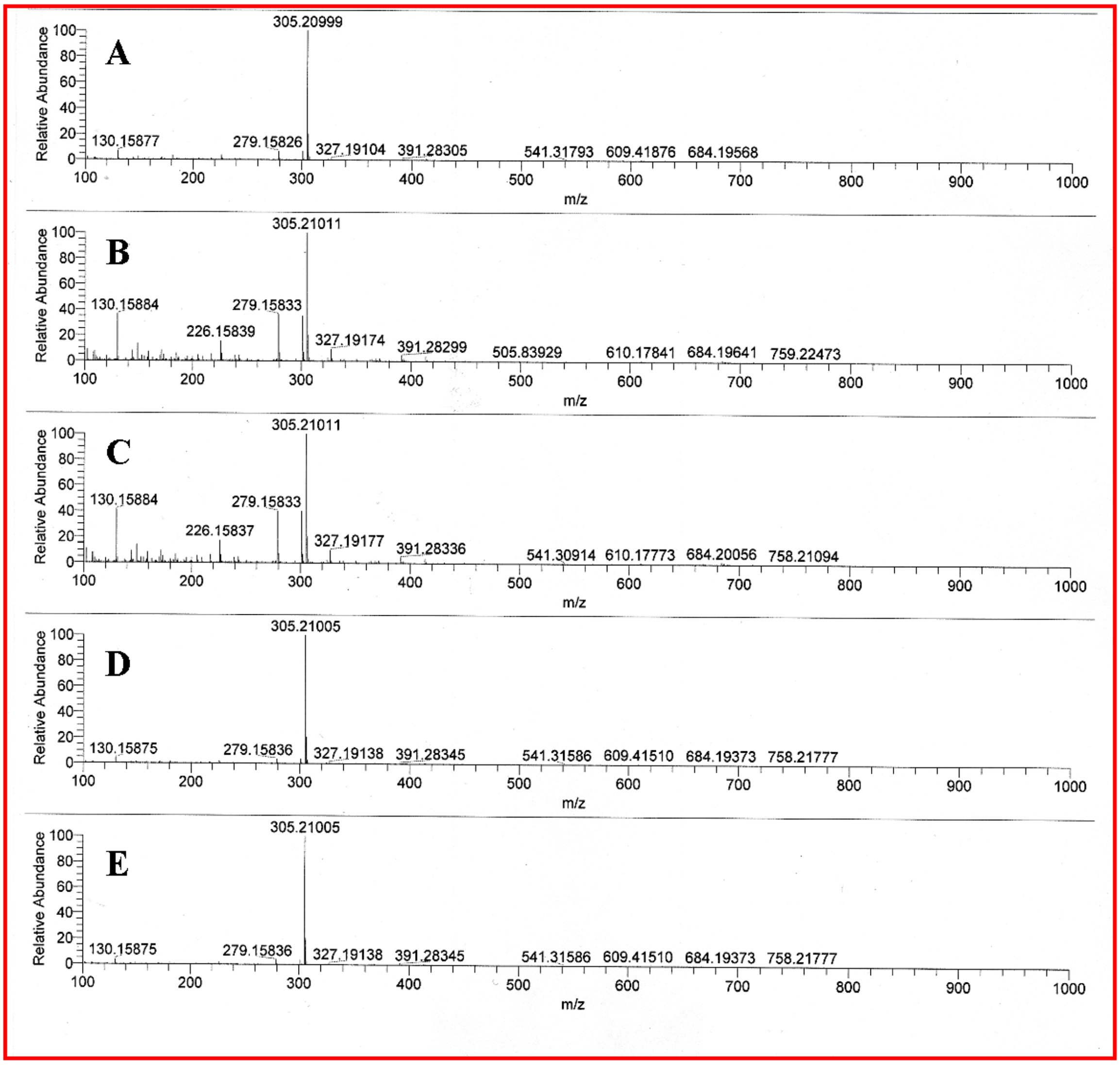

Figure S1. ESI mass spectra of metabolites 2 (**A**); 3 (**B**); 4 (**C**); 5 (**D**) and 6 (**E**) of testosterone produced by M01A82WS72I.

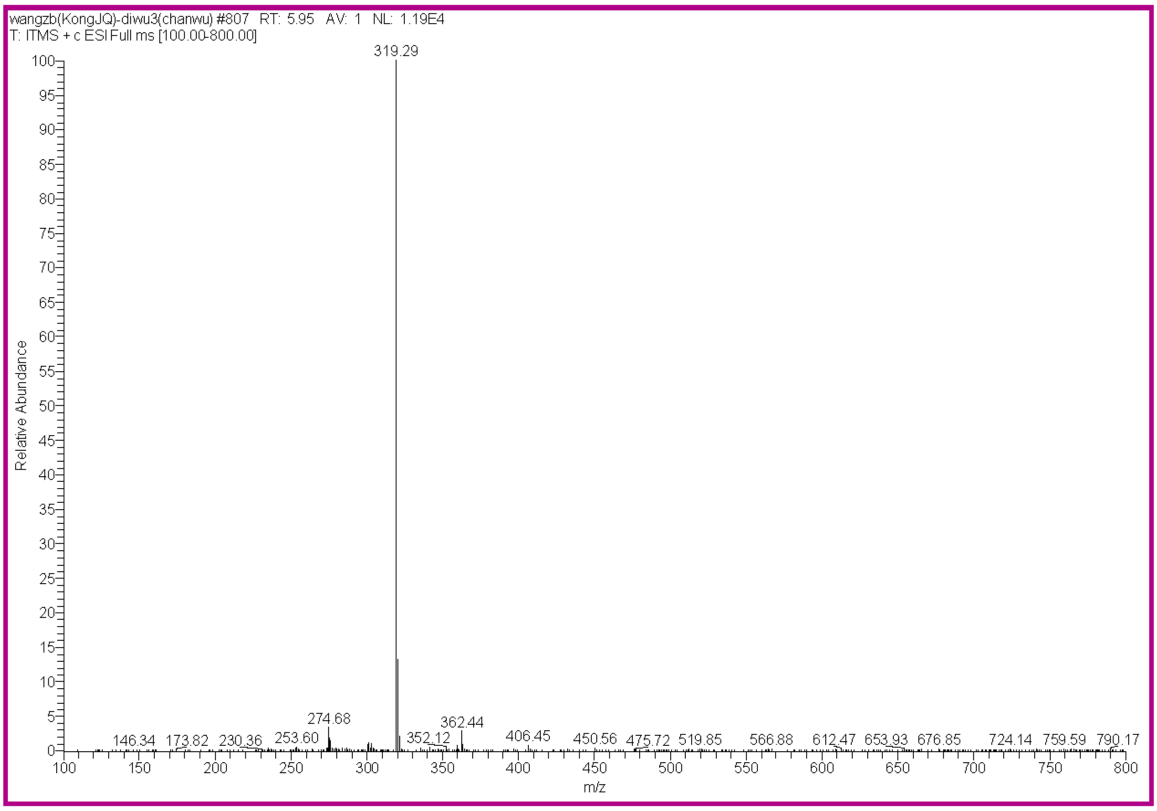

Figure S2. ESI mass spectra of 16β-OH-MT (metabolites 2) produced by M01A82W.

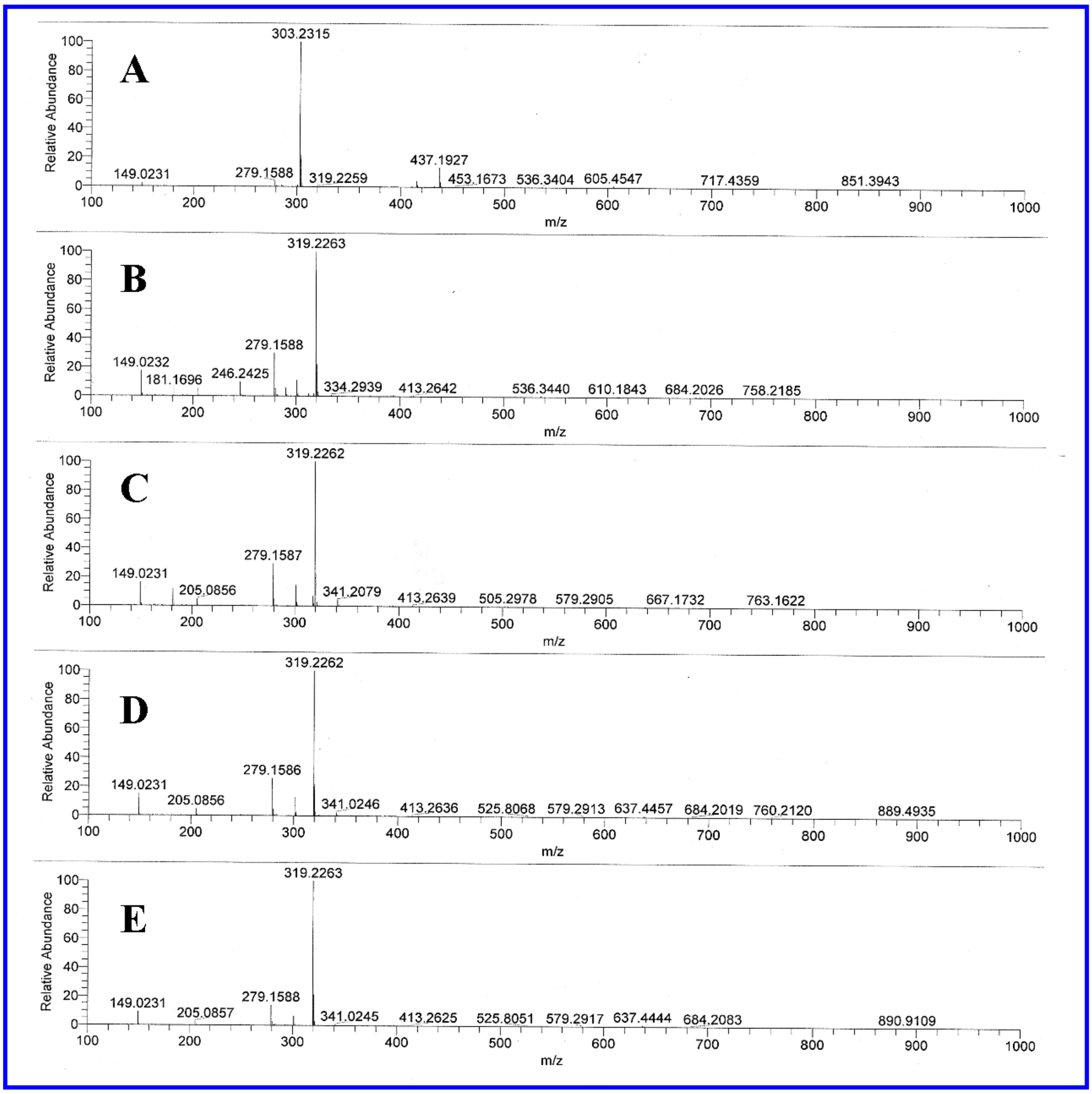

Figure S3. LC-MS analysis of methyltestosterone (**A**) and its monohydroxy metabolites (**B**–**E**) formed by P450 BM3 mutant pET28aS72I.

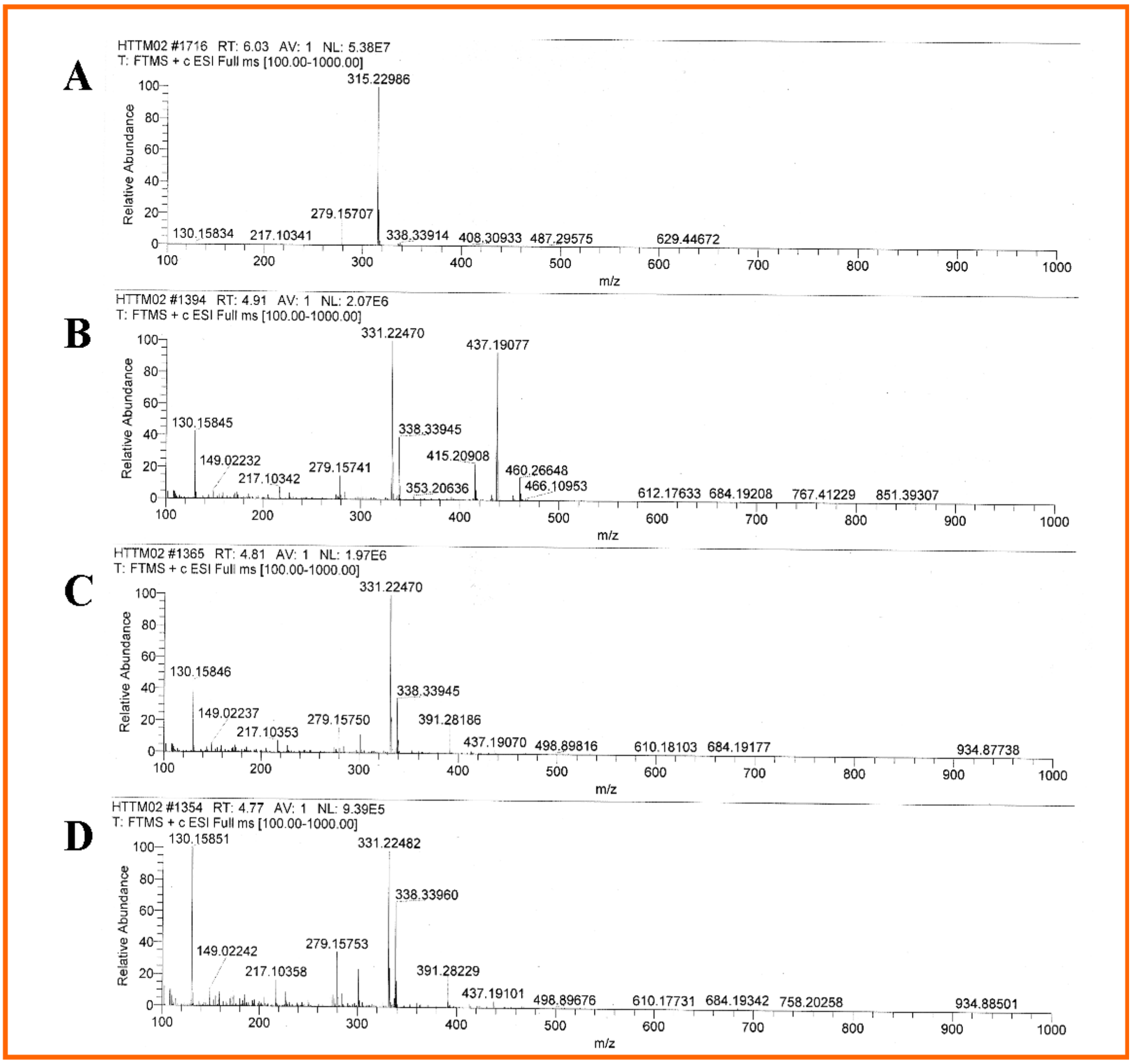

Figure S4. ESI mass spectra of progesterone (**A**) and its three monohydroxylated metabolites 2 (**B**); 3 (**C**) and 4 (**D**).

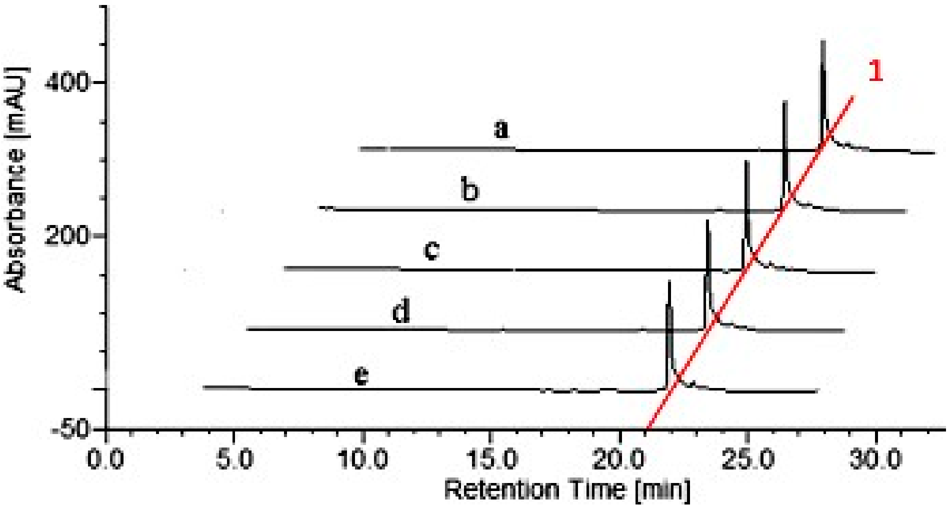

Figure S5. HPLC chromatograms of pregnenolone (**a**); pregnenolone incubations with empty vector (**b**); M01A82W (**c**); M01A82WS72I (**d**) and M11A82W (**e**). 1 shows pregnenolone standard.

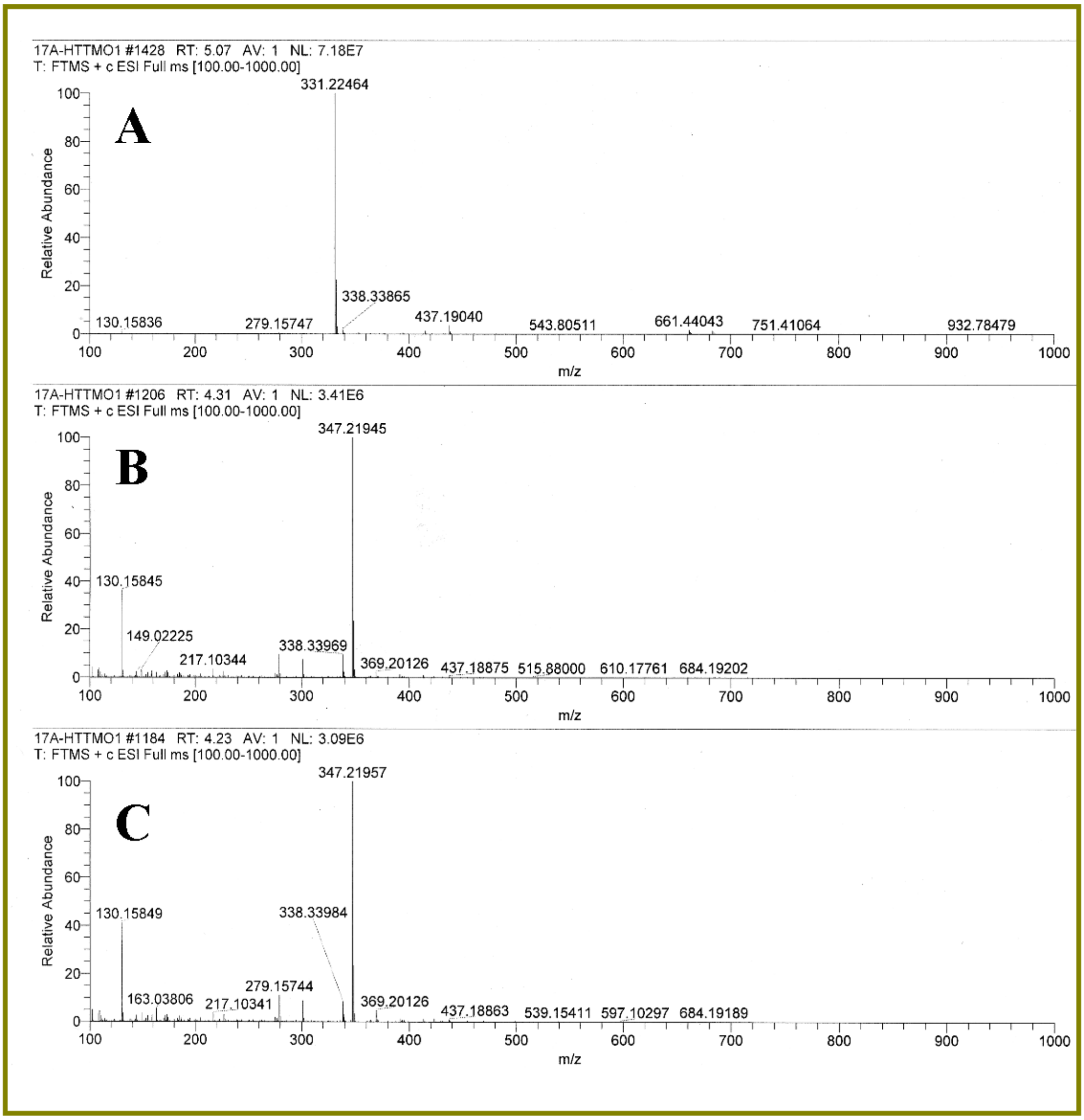

Figure S6. ESI mass spectra of 17α-hydroxyprogesterone (**A**) and its two monohydroxylated products 2 (**B**) and 3 (**C**).

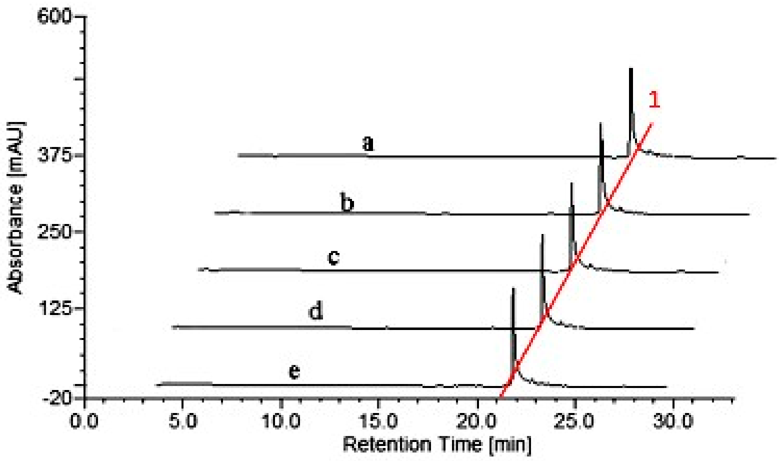

Figure S7. HPLC chromatograms of 17α-hydroxypregnenolone (**a**); 17α-hydroxypregnenolone incubations with empty vector (**b**); M01A82W (**c**); M01A82WS72I (**d**) and M11A82W (**e**). 1 shows 17α-hydroxypregnenolone standard.

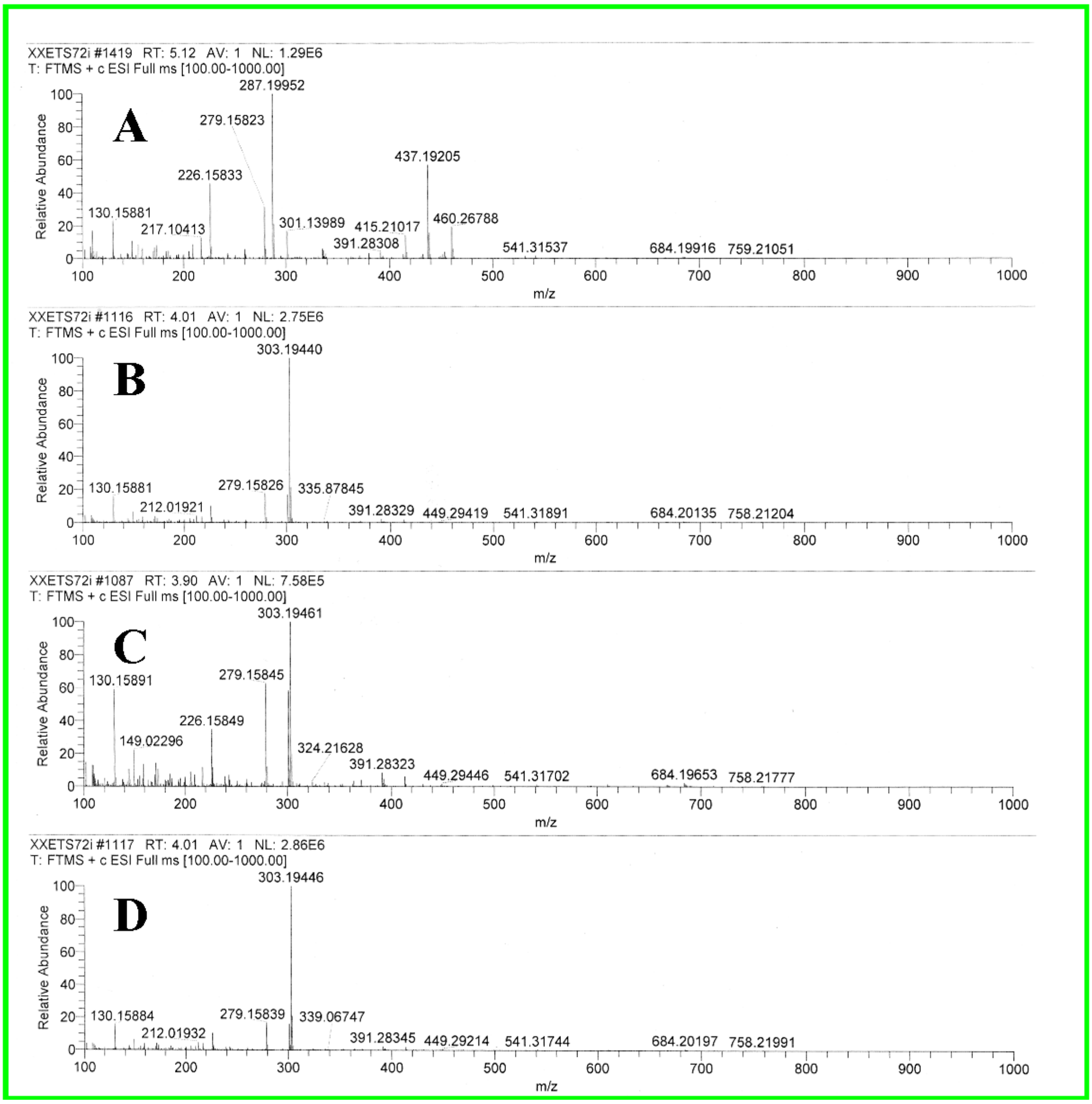

Figure S8. ESI mass spectra of androstenedione (**A**) and its three monohydroxylated metabolites 2 (**B**); 3 (**C**) and 4 (**D**).

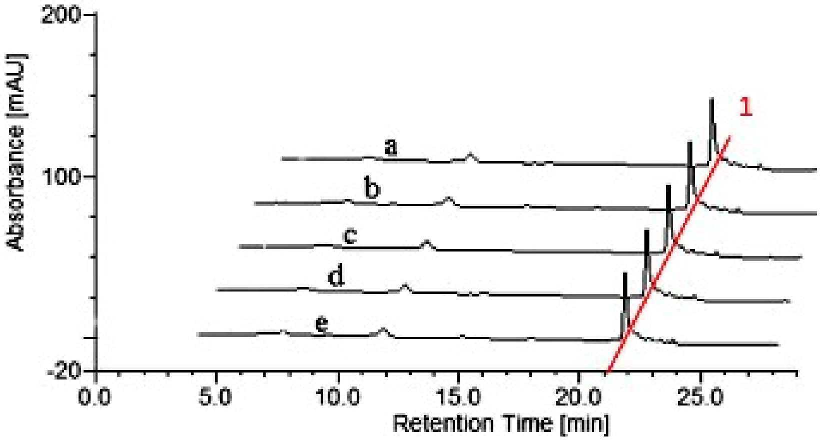

Figure S9. HPLC chromatograms of DHEA (**a**); DHEA incubations with empty vector (**b**); M01A82W (**c**); M01A82WS72I (**d**) and M11A82W (**e**). 1 shows DHEA standard.

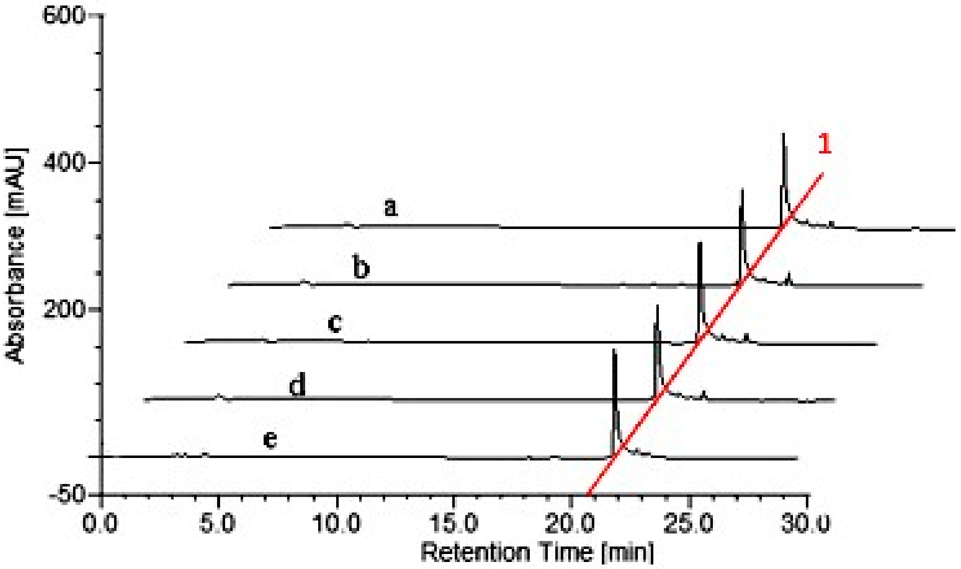

Figure S10. HPLC chromatograms of androstenediol (**a**); androstenediol incubations with empty vector (**b**); M01A82W (**c**); M01A82WS72I (**d**) and M11A82W (**e**). 1 shows androstenediol.
